# From incidental positron emission uptake to *in-vivo* phenotyping: a short history of positron emission tomography in the study of atherosclerosis and the vulnerable plaque

**DOI:** 10.3389/fcvm.2026.1767077

**Published:** 2026-07-08

**Authors:** Retesh Bajaj, Ming Young Simon Wan, Kris Thielemans, Eren Ozan Bakır, Soe Maung, Akash Sivananthan, Tom Crake, Anthony Mathur, Ryo Torii, Andreas Baumbach, Ashley Groves, Christos V. Bourantas

**Affiliations:** 1 Cardiology, Michael's Hospital, Toronto, ON, Canada; 2 Cardiology, University of Toronto, Toronto, ON, Canada; 3 Department of Radiology, University College London, London, United Kingdom; 4 Centre for Cardiovascular Medicine and Device Innovation, William Harvey Research Institute, Queen Mary University of London, London, United Kingdom; 5 Department of Cardiology, Barts Heart Centre, London, United Kingdom

**Keywords:** coronary atherosclerosis, CT coronary angiogram, positron emission tomography, vascular biology, vulnerable plaque

## Abstract

Positron emission tomography (PET) imaging has evolved over the last 20 years to a powerful tool for assessing vascular biology. The incidental uptake of PET tracers to the vessel wall provided the substrate for creating dedicated protocols and introducing novel agents for molecular imaging that targeted specific pathobiological mechanisms involved in the atherosclerotic process. Preliminary work with ^18^F-FDG underscored its efficacy to detect macrophage-rich inflammation, while, subsequent studies using ^18^F-NaF demonstrated its ability to detect active microcalcification and high-risk plaques. These encouraging results lead to the development of a new generation of tracers targeting macrophage receptors, chemokine signalling, angiogenesis, endothelial activation, and mitochondrial activity that have been validated against histology in animal models, endarterectomy, and autopsy tissue. This review provides an overview in the field describing the evolution of PET imaging in the study of atherosclerosis. It summarises the existing and emerging tracers, presents the biological processes that have been targeted by PET agents and collates the evidence from studies for vulnerable-plaque detection. Finally, it outlines technical hurdles and the current limitations of PET imaging that need to be addressed for clinical translation.

## Introduction

Positron emission tomography (PET) was conceived in the 1970s to detect glucose metabolism as a surrogate for visualising, quantifying and monitoring human physiology at a molecular level as a translational bridge between cell biology and clinical imaging, complementing, and at times transcending, purely anatomical imaging. Its earliest implementations combined transaxial coincidence detection with radiochemistry to measure regional glucose utilization and perfusion in tissues (1–3). Recent decades have seen PET mature from an instrument for quantifying regional metabolism and grow beyond into a niche clinical modality with the potential for phenotyping atherosclerotic activity in the arterial wall, empowering clinicians and researchers with an unprecedented tool for the study and management of atherosclerotic disease.

**Figure 1:**
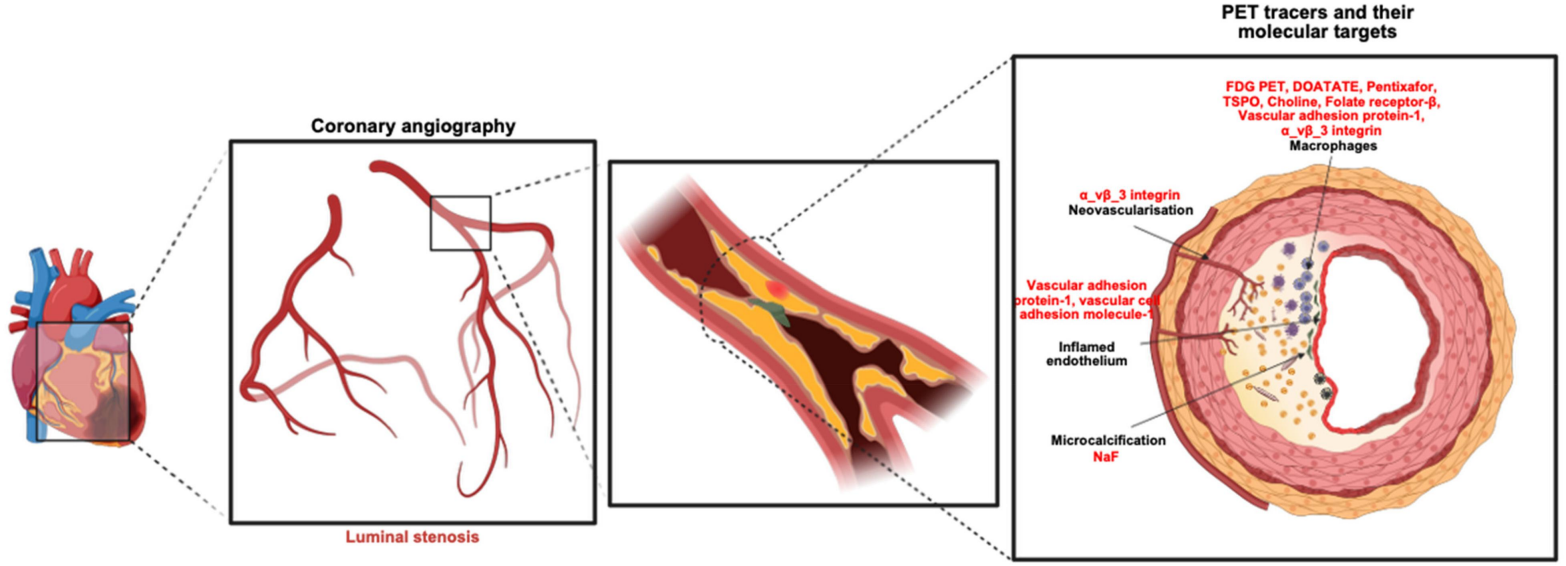
A representation of the potential molecular targets for PET tracers for studying atherosclerosis. Created in BioRender. Bajaj, R. (2026). https://BioRender.com/8yabdrf.

What started with the observation of 2-[^18^F]fluoro-2-deoxy-D-glucose (FDG) activity along the aorta and carotids on oncological scans, and then the first proof-of-concept studies demonstrating that FDG uptake co-localises with macrophage-rich carotid atheroma and correlates with histology (4, 5), grew into a comprehensive effort to phenotype the biology of atherosclerosis *in vivo*, extending beyond glycolysis to microcalcification, macrophage receptor expression, angiogenesis, chemokine signalling, and mitochondrial activation (Figure 1) (6, 7). These processes are instrumental in the formation and destabilization of atherosclerotic plaques. Although PET is well established in clinical practice for the diagnosis and staging of malignancy and for the assessment of selected systemic inflammatory disorders, its application in cardiovascular disease — and specifically in the *in-vivo* phenotyping of atherosclerosis — remains predominantly investigational, although this clinical positioning may evolve as the evidence base matures.

Over two decades, the field progressed through three broad phases. First, the FDG era established vascular PET's feasibility, reproducibility (especially in carotid and aortic beds), and links to risk. Second, the fluoride era re-purposed [^18^F] sodium fluoride [(^18^F)NaF] to image active microcalcification, enabling lesion-level coronary phenotyping and risk stratification. Third, a diversification of PET tracers expanded the palette beyond glycolysis and mineral to distinct hallmarks of plaque biology (8–11). This narrative review aims to provide an overview of the role of PET imaging in the study of coronary atherosclerosis by summarising tracers that have been investigated across the spectrum of development, from preclinical validation studies and histopathological correlations to first-in-human and clinical studies, with emphasis on their biological targets and translational challenges (Table 1).

**Table 1:** List of PET tracers for the assessment of atherosclerotic plaque (18).

Tracer (Isotope)	Biological target/mechanism	Pitfalls for coronary plaque assessment	Stage of evidence
^18^ **F-FDG**	Glucose metabolism in inflammatory cells (macrophages); plaque inflammation marker	- Very high physiological myocardial uptake; the protocols introduced to suppress myocardial uptake have a variable efficacy - The moving coronaries cause partial-volume and motion blurring	Prognostic/clinical outcome data
^18^ **F-NaF**	Binds to hydroxyapatite in microcalcifications; marker of active plaque mineralization	- There is rib/sternum and valvular uptake near the coronaries - The microcalcification signal is not always associated with vascular inflammation or plaque instability - The coronary motion reduces TBR and the accuracy in quantifying microcalcification activity	Prognostic/clinical outcome data
^64^ **Cu/** ^68^ **Ga-DOTATATE**	Somatostatin receptor-2 on activated macrophages (inflammation)	- There is an increased liver, spleen and kidney uptake which can complicate whole-field quantification. - The myocardial uptake is generally low but can increase in inflammatory cardiomyopathy, risking spill-in in select patients. - The detection of coronary uptake is limited by co-registration challenges due to motion and to the small coronary size.	Clinical observational data
^68^ **Ga-Pentixafor**	C-X-C chemokine receptor-4 (CXCR4) on inflammatory leukocytes (i.e., monocytes, macrophages)	- Strong spleen/bone-marrow signal adjacent to thoracic aorta. - The uptake in post-MI myocardium may confound coronary assessment. - CXCR4 expression is not macrophage-specific as it is also seen in hematopoietic cells and in endothelium.	Clinical observational data
**TSPO tracers** (e.g., ^18^F-FEDAC, ^18^PBR06)	18 kDa translocator protein (TSPO) on mitochondria of activated macrophages	- Low plaque-to-wall contrast; uptake may not exceed normal vessel wall. - High and variable myocardial signal with some TSPO ligands can result in spill-in, lowering coronary plaque contrast - potential defluorination for certain ^18^F-TSPOs, generating ^18F-fluoride with avid rib/sternal bone uptake that contaminates coronary signal; the extent is ligand-dependent (e.g., low for ^18F-PBR06, higher for others). - Binding is strongly genotype-dependent: the TSPO rs6971 (Ala147Thr) polymorphism creates high-, mixed-, and low-affinity binders; without genotyping/kinetic correction, inter-subject uptake varies 2–3×, biasing quantification. - TSPO is expressed in multiple vascular cell types (endothelium and smooth muscle, not just macrophages), which reduces specificity for macrophage-driven plaque inflammation. - TSPO in circulating blood cells yields relatively high blood-pool activity, further depressing coronary target-to-blood ratios.	Clinical observational data
^11^ **C/** ^18^ **F-Choline**	Phospholipid synthesis in cell membranes – marker of macrophage proliferation and metabolism.	- The physiologic uptake by the liver, pancreas and kidney is high resulting in scatter near the RCA. - There is some myocardial uptake that however is lower than the FDG	Clinical observational data
^18^ **Folate (** ^18^ **F-FOL)**	Folate receptor-*β* on activated macrophages	- There is a very high renal, urinary activity resulting in intense kidney, bladder signal	Preclinical evidence
^18^**F-4 V** (VCAM-1 ligand)	VCAM-1 – an endothelial adhesion molecule upregulated on inflamed endothelium	- VCAM-1 is upregulated in infarcted myocardium after MI. - Renal clearance and abdominal activity can affect inferior wall reconstruction	Preclinical evidence
^68^ **Ga-DOTA-Siglec-9**	Vascular adhesion protein-1 (VAP-1) synthesized by the inflamed endothelium also expressed by some macrophages	- High renal excretion resulting in kidneys and urine uptake. - The early blood-pool activity can obscure the activity in the small moving coronaries	First-in-human studies
**RGD peptides**(e.g., ^18^F-Galacto-RGD; ^18^Ga-RGD)	*α*_vβ_3 integrin – marker of angiogenesis expressed by neovasculature endothelial cells and by activated macrophages.	- There is a strong uptake in the healing myocardium post MI due to *α*_vβ_3 expression - The increased uptake in skeletal muscle and tendons and the variable bowel activity can add to the background signal - The integrin expression on activated leukocytes reduces endothelial specificity.	Clinical observational data

18 F-4 V, fluorine-18–labeled VCAM-1 ligand “4V”; ^18^F-FDG, fluorine-18 fluorodeoxyglucose; ^18^F-fluciclatide, fluorine-18 fluciclatide (αvβ3-binding RGD peptide); ^18^F-FOL, fluorine-18 folate (aluminum-fluoride–labeled folate); ^18^F-NaF, fluorine-18 sodium fluoride; ^64^Cu-DOTATATE, copper-64–labeled DOTATATE; ^68^Ga-DOTATATE, gallium-68–labeled DOTATATE; ^68^Ga-NODAGA-RGD, gallium-68–labeled NODAGA-RGD peptide; ^68^Ga-Pentixafor, gallium-68–labeled pentixafor (CXCR4 ligand); 89Zr-LA25, zirconium-89–labeled LA25 (human antibody Fab to OSE); apoE−/−, apolipoprotein E knockout; CCR2, C-C chemokine receptor type 2; CCR5, C-C chemokine receptor type 5; CXCR4, C-X-C chemokine receptor type 4; dOTA, 1,4,7,10-tetraazacyclododecane-1,4,7,10-tetraacetic acid; DOTATATE, DOTA-(Tyr^3^)-octreotate; FR-β, folate receptor-beta; MR, magnetic resonance; MRI, magnetic resonance imaging; NaF, sodium fluoride; NODAGA, 1,4,7-triazacyclononane-1-glutaric acid-4,7-acetic acid; OSE, oxidation-specific epitopes; PET, positron emission tomography; PET/CT, positron emission tomography/computed tomography; PET/MR, positron emission tomography/magnetic resonance; RGD, arginine-glycine-aspartate; SSTR2, somatostatin receptor subtype 2; TSPO, translocator protein (18 kDa); VAP-1, vascular adhesion protein-1; VCAM-1, vascular cell adhesion molecule-1.

## FDG-PET and vascular inflammation

### The first evidence supporting the value of FDG in assessing vascular biology

**Figure 2:**
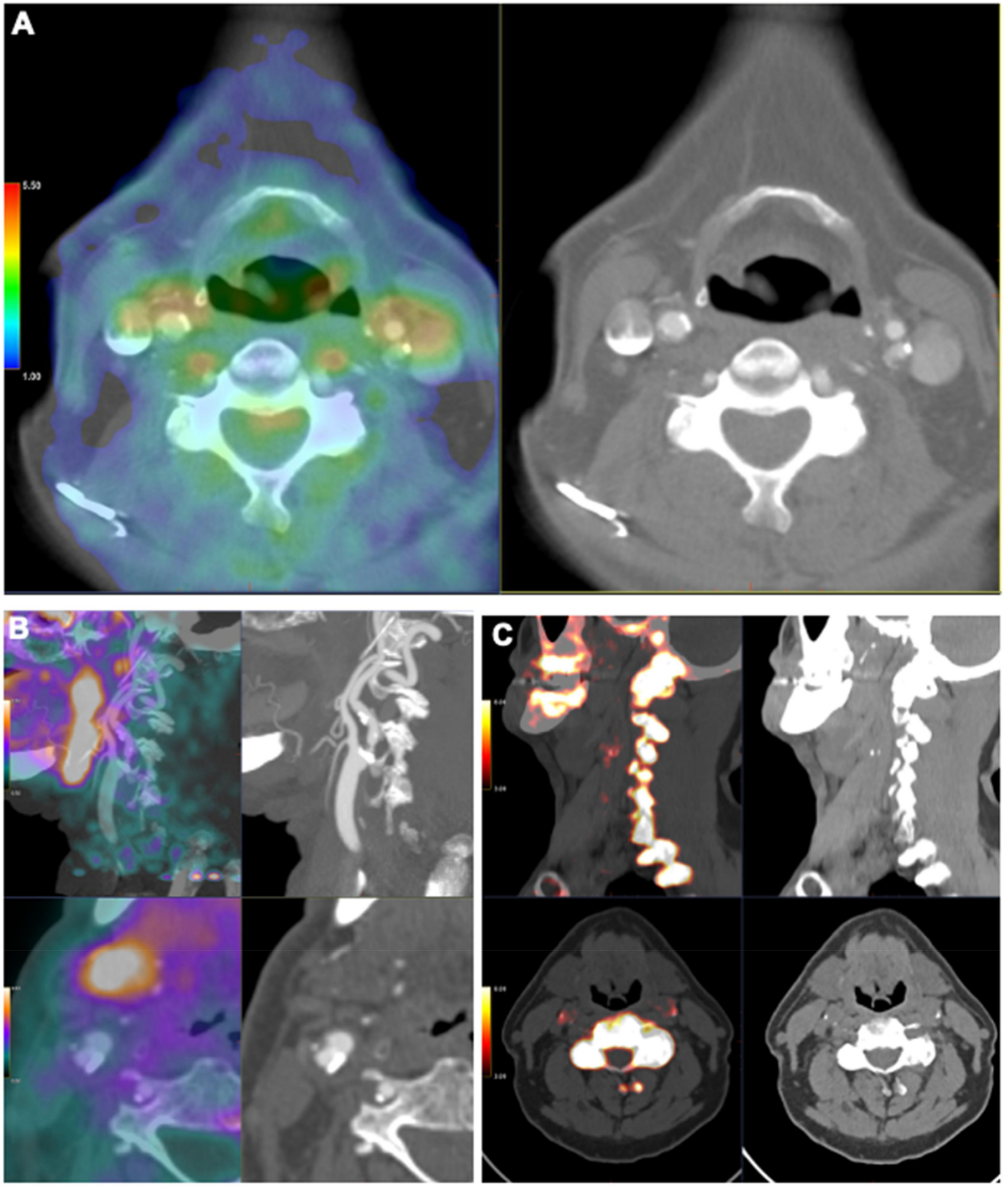
Representative figure demonstrating research examples of PET tracers used to study atherosclerotic plaque. **(A)**: the panel on the left shows used FDG PET/CT angiogram image showing higher activity at the left carotid plaque at the level of proximal internal carotid and on the right the corresponding CT angiogram image showing mixed calcified and non-calcified plaque components; **(B)**: left panels showing fused Ga^68^ DOTATATE PET/CT angiogram image showing higher activity at the right carotid plaque at the level of common carotid bifurcation; right panels showing corresponding CT angiogram image showing mixed calcified and non-calcified plaque components; **(C)**: left panels showing fused NaF PET/CT image showing focal tracer uptake localising specifically to areas of calcification visible on CT and right panels showing corresponding non contrast CT image showing foci of carotid wall calcification.

The first systematic observations of FDG uptake in arteries appeared in the early 2000s, with the work of Yun and colleagues (12) who noted arterial wall FDG uptake on routine scans, speculating that it may be explained by smooth muscle metabolism/proliferation or macrophage activity in atherosclerosis. The same group later showed that FDG PET uptake correlated explicitly with atherogenic risk factors: age and hypercholesterolaemia, suggesting its use in detecting atherosclerosis and its potential for monitoring disease progression (7). This was supported by a pre-clinical study reported by Lederman et al. (6), which demonstrated that PET detected with a fibre-optic catheter in a rabbit model of atherosclerosis could successfully distinguish atherosclerotic from normal arteries. Rudd et al. reported a landmark study in 2002 in 8 patients following cerebrovascular events showing FDG-PET activity to be 27% higher in affected carotid arteries with plaque events than the contralateral carotid arteries (Table 2, Figure 2), and demonstrated a histological correlation – accumulation of deoxyglucose in macrophage-rich areas of the plaque in endarterectomy samples from the carotid arteries studied *ex vivo* (4). This was the first evidence explicitly linking acute plaque events in human atheroma with FDG uptake, showing the potential of PET activity to go beyond anatomical assessment and detect biological processes associated with adverse clinical outcomes. This was supported by subsequent studies with hybrid PET/CT imaging, distinguishing PET activity from vascular calcification (13–15). These reports showed that PET and calcification rarely co-localised, suggesting that PET activity was a marker capturing a different disease stage to vascular calcification by CT. Further mechanistic insights came from an *ex vivo* study in 17 patients with severe carotid artery disease where the investigators found a significant correlation of FDG PET activity with macrophage presence on endarterectomy sections (r = 0.70; *p* < 0.0001), while the FDG activity did not correlate with plaque area or plaque thickness indicating that FDG activity was only related to plaque biology (5).

**Table 2:** Histological validation studies for tracers targeting atherosclerotic plaque progression and vulnerability.

PET Tracer	Study	Species/Model	Vessel Studied	Validation Method	Key Findings (effect sizes, p-values)
^18^F-FDG (fluorodeoxyglucose)	Rudd et al., 2002 (4)	Human (post-TIA carotid endarterectomy patients)	Carotid artery (plaques)	*Ex vivo* autoradiography and immunohistochemistry (CD68)	- FDG uptake was 27% higher in culprit carotid plaques vs the contralateral arteries (*p* < 0.05) - Autoradiography showed co-localization of FDG with macrophage-rich areas in the plaques
	Ogawa et al., 2004 (93)	Atherosclerotic WHHL rabbit models and a control group	Aorta	*Ex vivo* gamma counting, autoradiography & IHC	- PET imaging confirmed intense aortic FDG in atherosclerotic aortas of WHHL rabbits vs minimal uptake in the control group (DUR 1.47 ± 0.90 vs 0.44 ± 0.15, *p* < 0.01). - FDG uptake was strongly correlated with plaque macrophage count (r = 0.81).
	Tawakol et al., 2006 (5)	Human	Carotid artery	*In vivo* PET vs. *ex vivo* histology (CD68 IHC)	- Significant correlation was noted between PET FDG uptake and plaque macrophage content (r = 0.70, *p* < 0.0001) - No correlation was found between FDG uptake and plaque size/thickness, indicating that FDG reflects inflammation specifically
	Zhang et al., 2006 (94)	Rabbits	Aorta	*Ex vivo* autoradiography & histology	- FDG uptake-to-blood ratio correlated with macrophage area (r = 0.80, *p* < 0.0001).
	Calcagno et al., 2008 (95)	Rabbits	Aorta	PET with histology (micro-vessel count)	- FDG SUV showed a positive but non-significant correlation with neo-vessel count (r = 0.50, *p* = 0.103).
	Hyafil et al., 2009 (96)	Rabbits	Aorta	PET with histology (macrophage count)	- FDG SUV was higher in atherosclerotic compared to the control aortas (0.61 ± 0.12 vs 0.21 ± 0.02, *p* < 0.05). - FDG uptake correlated with macrophage density (r = 0.63, *p* < 0.001).
	Zhao et al., 2013 (97)	Rabbit aorta with triggered thrombosis	Aorta	PET with histology	- Vulnerable vs stable plaques had higher pre-trigger FDG (SUVmean 1.097 ± 0.189 vs 0.768 ± 0.111, *p* < 0.001). - Macrophage count correlated with FDG (r = 0.316, *p* < 0.001).
	Tarkin et al., 2015 (98)	Porcine *ex vivo* models	Coronary arteries	*Ex vivo* autoradiography & histology	- FDG was higher in diseased vs normal vessel wall (intimal thickening 1.7 ± 0.7×, *p* = 0.004; atheroma 4.1 ± 2.3×, *p* = 0.003). - Vessel-to-blood ratio was higher in atheroma vs disease-free-segments (2.6 ± 1.2 vs 1.3 ± 0.7, *p* = 0.04).
^11^C-Choline/^18^F-fluorocholine	Laitinen et al., 2010 (57)	Mouse LDLR⁻/⁻ ApoB100/100 hyperlipidemic model and a control group	Ascending aorta and arch	*Ex vivo* autoradiography and immunohistochemistry	- Atherosclerotic aortas showed ∼1.9× higher ^11^C-choline uptake than the control group (*p* = 0.0016) - ^11^C-choline uptake was significantly higher in macrophage-rich regions compared to the normal wall (plaque to healthy vessel wall ratio 2.3 ± 0.6, *p* = 0.014). - ^11^C-choline uptake was higher in inflamed (macrophage-rich) vs non-inflamed plaque areas (2.55 ± 0.78 vs 1.41 ± 0.48, *p* < 0.001) suggesting macrophage-mediated tracer accumulation
	Matter et al., 2006 (99)	ApoE⁻/⁻ mice	Aorta	*Ex vivo* autoradiography & histology	- ^18^F-fluorocholine uptake correlated with lipid area on Oil-Red-O (r = 0.842, *p* < 0.0001) and with CD68-positive macrophage areas (r = 0.740, *p* < 0.0001) - ^18^F-fluorocholine detected plaques with a sensitivity of 84% - ^18^F-fluorocholine identified more plaques than ^18^F-FDG in this model (correlation with lipid area: r = 0.261, *p* < 0.05 and sensitivity for plaques 64%).
	Hellberg et al., 2016 (100)	LDLR⁻/⁻ ApoB100/100 in mice with and without T2DM	Aorta	PET and *ex vivo* autoradiography & histology	- ^18^F-fluoromethylcholine signal was increased in the atherosclerotic plaques compared to the wall (240 ± 20 vs 89 ± 6.8 PSL/mm^2^, *p* < 0.001) with a plaque-to-wall ratio 2.6 ± 0.085 - Macrophage-rich plaque areas had a higher uptake than the low-macrophage areas (110 ± 5.3 vs 81 ± 4.5 PSL/mm^2^, *p* = 0.0027) - Aorta-to-myocardium contrast was higher than with ^18^F-FDG in diabetic mice (8.4 ± 0.80 vs 6.5 ± 0.80, *p* < 0.001).
^11^C-PK11195	Laitinen et al., 2009 (60)	Mouse LDLR⁻/⁻ ApoB100/100 models and a control group	Aorta	*In vitro* ^3^H-PK11195 binding assay; *ex vivo* ^11^C- PK11195 autoradiography and histology	− Whole-aorta uptake was higher in the LDLR⁻/⁻ ApoB100/100 models than the controls (4.11 ± 1.13 vs 2.21 ± 0.65%IA/g, *p* = 0.0016). - Plaque-to-healthy wall ratio was 2.3 ± 0.6 (*p* = 0.014) - Macrophage-rich (Mac-3⁺) regions showed greater uptake than non-inflamed regions (2.55 ± 0.78 vs 1.41 ± 0.48, *p* < 0.001).
^18^F-FEDAC (TSPO)	Hellberg et al., 2017	LDLR⁻/⁻ApoB100/100 and healthy mice	Aorta	*Ex vivo* autoradiography and IHC (macrophages/TSPO); *in-vitro* blocking.	- Whole-aorta radioactivity was 1.3× higher in atherosclerotic vs healthy mice (*p* = 0.028). - Plaque areas containing macrophages had greater uptake than macrophage-negative regions (190 ± 54 vs 40 ± 13 PSL/mm^2^, *p* < 0.001) - *in-vivo* aortic uptake did not exceed healthy wall, indicating substantially non-specific wall signal.
	Zhang et al., 2019	ApoE⁻/⁻ mice and (wild-type) C57BL/6 J mice	Aorta	*In-vivo* micro-PET/CT; ex-vivo immunofluorescence co-staining (CD68, F4/80)	- The aortic uptake was higher in ApoE⁻/⁻ vs wild-type at 22 weeks (9.04 ± 1.71 vs 6.37 ± 0.45, *p* < 0.05) - The plaque-to-muscle ratio was increased at the 32 compared to the 22 week (15.46 ± 4.20 vs 8.09 ± 1.12, *p* < 0.05) - PET signal co-localized with macrophage markers and TSPO on histology
	Maekawa et al., 2021 (101)	High-cholesterol diet rabbit undergoing balloon injury; human coronary plaques (autopsy and PCI aspirates)	Rabbit carotid and human coronaries	*In vivo* rabbit PET/CT; *ex vivo* autoradiography and TSPO IHC (rabbit & human)	- In the injured carotids the SUV was higher compared to the control groups (0.574 ± 0.24 vs 0.277 ± 0.13, *p* < 0.05) - Autoradiography intensity correlated with macrophages (r≈0.64) and TSPO (r≈0.67) - TSPO expression was higher in AMI cases and in vulnerable vs early/stable lesions; TSPO localized to macrophages in all aspirated thrombotic plaques
^18^F/^64^Cu/^68^Ga-DOTATATE (SSTR2)	Tarkin et al., 2017 (10)	Patients undergoing carotid endarterectomy	Carotid arteries	*In vivo* PET/CT (head/neck); *ex vivo* autoradiography and SSTR2 IHC on excised plaques	- Increased ^68^Ga-DOTATATE uptake was localized to CD68 macrophage-rich areas of plaques, corresponding to SSTR2 expression - There was a strong correlation between SSTR2 mRNA and *in-vivo* ^68^Ga-DOTATATE uptake (r = 0.89, *p* = 0.02).
	Wan et al., 2019	Humans undergoing carotid endarterectomy after recent events	Carotid arteries	*In-vivo* ^68^Ga-DOTATATE PET/CT; ex-vivo SST2 immunohistochemistry on excised plaques.	- No difference in any PET metric was noted between symptomatic and contralateral carotids; all SUV/TBR p-values were >0.10 - The excised plaques showed no SSTR2 membrane expression immunohistochemistry suggesting that the DOTATATE activity did not reflect receptor-mediated plaque uptake in this cohort
	Toner et al., 2022 (102)	Mice and rabbits	Aorta	*In vivo* PET; *ex vivo* validation	- In atherosclerotic mice the aortic ^64^Cu-DOTATATE SUVmax was higher compared to healthy controls [1.1 (0.9–1.3) vs 0.5 (0.5–0.6), *p* = 0.0286] - In atherosclerotic rabbits the aortic ^68^Ga-DOTATATE SUVmax was higher compared to the healthy controls [0.415 (0.338–0.499) vs 0.253 (0.197–0.285), *p* = 0.0002). - Macrophage burden on *ex vivo* assays were increased in atherosclerotic rabbits compared to controls and this aligned with the increased DOTATATE tracer signal. The FDG signal that was used as a comparator was also increased but showed only a weak correlation with the DOTATATE uptake (r = 0.352).
	Grandjean et al., 2023 (103)	Rabbits with and without diet-induced atherosclerosis	Aorta and peripheral arteries	*In vivo* PET/CT; *ex vivo* autoradiography, gene expression and IHC	- Atherosclerotic rabbits demonstrated significantly higher ^64^Cu-DOTATATE uptake in the vessel wall compared to healthy controls (SUVmean: 2.30 ± 0.27 vs 1.65 ± 0.16, *p* = 0.047). - ^64^Cu-DOTATATE tracer uptake correlated with the presence of macrophages on histological staining in atherosclerotic rabbit plaques and had a significantly higher absolute plaque uptake than the FDG and ^18^F-NaF (SUVmean for ^64^Cu-DOTATATE 2.29 vs 1.50 for FDG, *p* = 0.001 and vs 1.55 for ^18^F-NaF; *p* = 0.003). - No cross-correlation was observed between FDG, ^18^F-NaF, and ^64^Cu-DOTATATE signals, underlining a distinct target specificity.
^68^Ga-Pentixafor (CXCR4)	Hyafil et al., 2017 (104)	Rabbit on HFD undergoing carotid endothelial injury	Carotid artery, aorta	*In vivo* PET/MR; *ex vivo* ^125^I-Pentixafor autoradiography and IHC	- The tracer's uptake in aortic plaques of HFD rabbits with endothelial injury was higher than the control group (mean TBR 1.95 ± 0.51 vs 1.22 ± 0.25, *p* < 0.05). - Autoradiography confirmed that this localized to macrophage-rich regions; the uptake correlated with CXCR4 IHC (r^2^ = 0.61, *p* < 0.05)
	Li et al., 2018	Oncology patients undergoing PET/MR and patients with symptomatic carotid stenosis listed for TEA	Carotid arteries	*In-vivo* PET/MR; ex-vivo endarterectomy histology/IHC showing CXCR4 and CD68 co-localization in plaques.	- Eccentric carotid plaques showed a higher ^68^Ga-Pentixafor uptake than non-eccentric plaque segments (non-eccentric vs severely eccentric 1.55 vs 1.29, *p* ≤ 0.05) - Histology confirmed CXCR4 and CD68 co-localization in inflamed atheromas
	Baba et al., 2021 (105)	ApoE⁻/⁻ mice model of atherosclerosis regression; high fat diet followed by adenoassociated virus 8 vector encoding murine ApoE treatment	Aorta	*In vivo* PET imaging using the ^64^Cu-DOTA-vMIP-II tracer and histology	- CXCR4 PET tracked endothelial injury and monocyte recruitment during plaque progression - During atherosclerosis regression, PET signal fell significantly at the same timepoint with the reduction in monocyte recruitment that preceded the reduction in macrophage burden – suggesting that the tracer specifically targets monocytes recruitment - Pharmacological CXCR4 blockade and endothelial-specific CXCR4 deletion both significantly lowered PET signal (*p* < 0.01), supporting specificity of tracer for CXCR4.
^18^F-NaF (sodium fluoride)	Youn et al., 2020 (48)	*Ex vivo* coronary plaques from 18 human hearts	Coronary arteries	*Ex vivo* PET/CT of excised arteries with histology for microcalcification, plaque type, and macrophage presence.	- Micro-calcified plaques showed high NaF uptake (mean TBR 9.0 ± 9.7) vs plaques without microcalcification (TBR 2.9 ± 3.8, *p* < 0.0001). - Advanced fibroatheromas had a greater uptake than early lesions (TBR 10.7 ± 10.3 vs 6.1 ± 8.4 in pathological intimal thickening, *p* = 0.004). - No correlation was noted between tracer's uptake and CD68 staining
	Irkle et al., 2015 (44)	Human carotid endarterectomy (*ex vivo*)	Carotid plaques	*In-vitro* binding assays; *ex vivo* plaque analysis	- Microcalcifications had a higher fluoride adsorption than macrocalcifications (0.59 ± 0.23 vs 0.37 ± 0.15, *p* < 0.02). - The tracer had a high affinity constant (K_D_ 0.6 pM) showing high sensitivity for hydroxyapatite.
	Moss et al., 2020 (106)	Human coronary autopsy (*ex vivo*)	Coronary arteries	*Ex vivo* specimens incubated with ^18^F-NaF then micro-PET/CT; co-registered micro-CT with histology	- Plaque ^18^F-NaF activity was 10 times higher than the activity of the myocardium (median 157.5 vs 14.9 kBq/mL, *p* < 0.001) - Uptake was higher in plaques without macrocalcification (158.1 vs 149.8 kBq/mL, *p* = 0.047). - High-intensity uptake co-localised with microcalcification and hydroxyapatite (qualitative histology; authors do not provide measurements) - Regions with high ^18^F-NaF co-localised with osteopontin and RUNX-2 staining—markers of active osteogenic mineralisation: Osteopontin-positive plaques had higher activity than osteopontin-negative (mean 145.0 vs 94.5 kBq/mL; *p* < 0.0001). RUNX-2-positive also higher (139.8 vs 102.6 kBq/mL; *p* = 0.0043). - The signal localised particularly to the intima and minimally in the media
	Nogales et al., 2021 (107)	Minipig with familial hypercholesterolemia or prolonged high-fat diet	Aorta, iliac, and coronary arteries	*In vivo* PET-CT; *ex vivo* PET-CT of extracted arteries, autoradiography and histology	- There was a co-localization of the ^18^F-NaF with histological calcifications in all the examined arteries (r = 0.69, *p* < 0.001) - There was no association between the tracer's uptake and macrophage area (r = 0.05, *p* = 0.66). - ^18^F-NaF uptake correlated more with macro- than microcalcification (r = 0.55 vs r = 0.33; both *p* < 0.001).
	Wen et al., 2023	Patients undergoing CABG with adjunctive coronary endarterectomy	Coronary arteries	*In-vivo* PET-CT; ex-vivo histology and immunohistochemistry (vulnerability features, CD68; inflammatory cytokines).	- High-risk plaques had a higher *in-vivo* ^18^F-NaF activity [median TBR 1.88 (1.41-2.54) vs 1.12 (0.91–1.54), *p* < 0.001] - The AUC of the tracer to detect high-risk plaques was 0.80 - ^18^F-NaF activity correlated with CD68 staining and inflammatory cytokines (all *p* < 0.05), but not osteogenic markers (staining with osteopontin, RUNX-2, osteocalcin; all *p* > 0.05)
^18^F-fluciclatide (RGD)	Jenkins et al., 2019 (11)	*Ex vivo* human carotid endarterectomy plaques; *in vivo* thoracic aorta PET/CT in patients with stable vs recent MI.	Carotid arteries	*Ex vivo* plaque autoradiography compared with immunohistochemistry; *in vivo* PET correlations with CT-derived plaque metrics.	- ^18^F-fluciclatide binding was found in regions of active plaque angiogenesis (correlation with aortic wall thickness r = 0.57, *p* = 0.001, with plaque volume r = 0.56, *p* = 0.001 and with CT calcium score r = 0.37, *p* = 0.01) - Autoradiography of excised carotid plaques showed intense tracer uptake in ruptured/high-risk areas, with minimal binding in adjacent normal vessel segments - The aortic uptake was higher in patients with a MI than those with stable coronary artery disease (TBRmax 1.29 vs 1.21, *p* = 0.02).
RGD peptides (αvβ3)	Laitinen et al., 2009 (108)	LDLR(−/−)ApoB(100/100) atherosclerotic mice	Aorta	*Ex vivo* plaque autoradiography with receptor blocking; histology and immunohistochemistry	- Focal ^18^F-Galacto-RGD uptake was higher in plaques versus adjacent normal wall/adventitia on autoradiography (55 ± 10 PSL/mm^2^ vs 39 ± 6 PSL/mm^2^ for the normal wall and 37 ± 7 PSL/mm^2^ for the adventitia, *p* ≤ 0.001 for both) - Tracer's uptake was associated with macrophage density (*p* = 0.003), but not with the connective tissue (*p* = 0.727) or lipid cores (*p* = 0.937)
	Saraste et al., 2012 (109)	LDLR(−/−)ApoB(100/100) atherosclerotic mice randomized to continue high-fat diet vs diet intervention	Aorta	*In-vivo* PET; ex-vivo autoradiography & histology	- Aortic ^18^F-Galacto-RGD uptake was lower with diet intervention vs high-fat diet (0.16 vs 0.23%ID/g, *p* < 0.01) - The plaque autoradiography signal was 35% lower with diet intervention (*p* = 0.007). - Plaque ^18^F-Galacto-RGD uptake correlated with tritiated deoxyglucose uptake and with nuclear density (both *p* = 0.01)
^18^F-AlF-Folate (“18F-FOL”)	Silvola et al., 2018 (74)	Atherosclerotic mouse (LDLR(−/−) ApoB (100/100) models; rabbit models on atherosclerosis diet; human carotid endarterectomy sections	Aorta (mice, rabbits); Carotid plaques (humans)	*In vivo* PET/CT in animals; *ex vivo* autoradiography and immunohistochemistry in animals & human tissues; folate-blocking specificity tests.	- In mice, aortic arch maximum TBR was higher in diseased vs the control group (1.5 ± 0.34 vs 0.71 ± 0.18, *p* < 0.001) - In rabbits, plaque uptake correlated with macrophage density on histology and showed plaque-to-background ratios comparable to FDG while the myocardial uptake was lower than FDG - In human carotid sections, folate blocking reduced binding by 88 ± 8.9%, confirming specificity.
	Müller et al., 2014 (110)	Human carotid endarterectomy	Carotid plaques	*Ex vivo* sections studied with autoradiography and immunohistochemistry	- 3′-aza-2′-^18^F-fluorofolic acid showed 7.7-fold higher binding in plaque vs normal arteries (*p* = 0.049) - Tracer binding co-localized with FR-β–positive and CD68-positive macrophages on immunohistochemistry.
VCAM-1 ligands	Bala et al., 2016 (111)	ApoE⁻/⁻ mice	Aorta	*In vivo* PET/CT; *ex vivo* autoradiography and immunohistochemistry	- ^18^F-FB-cAbVCAM1-5 tracer uptake increased with the histological high-risk plaque score (r^2^ = 0.675, *p* < 0.001). - The tracer showed high contrast for plaque detection due to low myocardial uptake (lesion-to-myocardium uptake ratio of 12.4 ± 0.4, lesion-to-blood uptake ratio 3.3 ± 0.4).
	Senders et al., 2019 (112)	ApoE⁻/⁻ mice and rabbits	Aorta	Three ^64^Cu-labeled nanobodies against VCAM-1, lectin-like oxidized low-density lipoprotein receptor (LOX)-1, and macrophage mannose receptor (MMR) were screened by *in-vivo* PET/MRI with ex-vivo autoradiography and immunohistochemistry	- MMR showed a higher aortic wall to blood ratio (SUVmax medians MMR 3.3 vs 1.0 for VCAM-1 1.0 vs 1.7 for LOX-1) - MMR uptake was high in the most disease segment compared to VCAM-1 and LOX-1 (SUVmax medians for the uptake in the most diseased segment vs the myocardium 3.2 for the MMR vs 4.0 for the VCAM 1 vs 2.5 for LOX-1). - Autoradiography confirmed that the MMR tracer had four times higher counts in the most-diseased segment vs the least-diseased segments. For the VCAM the counts were three times higher and for the LOX-1 the counts were one and half higher in the most diseased segments compared to the least disease segment. - MMR uptake correlated with vessel-wall area r = 0.55 (*p* < 0.001) in a plaque progression model
	Bridoux et al., 2020 (113)	ApoE⁻/⁻ mice	Aorta	VCAM-1 nanobody PET study; *in-vivo* PET/CT imaging with a competitive receptor-blocking control to prove VCAM-1–specific binding; *ex vivo* histology	- The aortic uptake was 2.15 ± 0.06 higher compared to VCAM-1 receptor blocked controls (*p* < 0.03)
VAP-1 (^68^Ga-/^18^F-Siglec-9)	Silvola et al., 2016 (79)	Atherosclerotic mouse (LDLR(−/−) ApoB (100/100) models and patients undergoing carotid endarterectomy	Aorta (mouse models); Carotid (humans)	*In vivo* PET/CT with VAP-1 receptor competitive blocking; *ex vivo* immunohistochemistry on carotid endarterectomy histology.	- Whole-aorta uptake was higher in atherosclerotic vs the control mice (0.83 ± 0.33 vs 0.46 ± 0.10%IA/g; *p* = 0.001) - VAP-1 receptor blockade reduced plaque autoradiography signal by 48% (*p* = 0.026) - Plaque signal correlated with macrophage density (r = 0.58, *p* = 0.022) - In carotid endarterectomy samples immunohistochemistry revealed that VAP-1 localized to endothelial cells of intraplaque neovessels and co-localized with biotinylated Siglec-9 in carotid endarterectomy sections.

18 F-AlF-Folate, fluorine-18 aluminum-fluoride–labeled folate; ^18^F-FDG, fluorine-18 fluorodeoxyglucose; ^18^F-FEDAC, fluorine-18 fluoroethyl–DPA-714 analog (TSPO ligand); ^18^F-fluciclatide, fluorine-18 fluciclatide; ^18^F-FOL, fluorine-18 folate; ^18^F-NaF, fluorine-18 sodium fluoride; ^64^Cu-DOTATATE, copper-64–labeled DOTATATE; ^68^Ga-DOTATATE, gallium-68–labeled DOTATATE; ^68^Ga-pentixafor, gallium-68–labeled pentixafor; AMD3100, plerixafor (CXCR4 antagonist); ApoB100/100, apolipoprotein B 100/100 knock-in; apoE−/−, apolipoprotein E knockout; CAD, coronary artery disease; CD31, cluster of differentiation 31; CD68, cluster of differentiation 68; CT, computed tomography; CXCR4, C-X-C chemokine receptor type 4; DOTA, 1,4,7,10-tetraazacyclododecane-1,4,7,10-tetraacetic acid; DOTATATE, DOTA-(Tyr^3)-octreotate; DPA-714, TSPO ligand DPA-714; DUR, differential uptake ratio; FDG, fluorodeoxyglucose; FR-β, folate receptor-beta; IHC, immunohistochemistry; MI, myocardial infarction; MRI, magnetic resonance imaging; NaF, sodium fluoride; PET, positron emission tomography; PET/CT, positron emission tomography/computed tomography; PET/MR, positron emission tomography/magnetic resonance; PMID, PubMed identifier; PPV, positive predictive value; RGD, arginine-glycine-aspartate; SUV, standardized uptake value; SUVmean, mean standardized uptake value; SSTR2, somatostatin receptor subtype 2; TBR, target-to-background ratio; TBRmax, maximum target-to-background ratio; TIA, transient ischemic attack; TSPO, translocator protein (18 kDa); WHHL, watanabe heritable hyperlipidemic (rabbit).

Paulmier et al. were the first that showed an association between PET uptake in arteries and future cardiovascular events; in this report, standardised uptake value (SUV) was used to quantify PET activity (16, 17). Since then several other studies have confirm a link between FDG activity and cardiovascular risk (18–22). Tahara et al. were among the first to examine the dynamic nature of PET activity when they reported that simvastatin appeared to reduce plaque FDG activity (but not dietary changes alone); this was followed by Tawakol et al. in 2013 (23) who reported that following statin intensification, FDG activity was reduced in a dose-dependent manner, without correlation with lipid profile changes. These findings highlighted for the first time the potential value of FDG activity as a surrogate endpoint to study the efficacy of novel pharmacotherapies targeting vascular inflammation (23–28).

### Challenges of FDG imaging in the study of coronary atherosclerosis and engineering solutions

The first application of FDG in the study of coronary artery disease and vulnerable plaque detection was reported in 2010 by Rogers et al. who found an increased FDG activity in culprit coronary lesions in patients with an acute coronary syndrome (ACS) compared to non-ACS stented segments and stable angina patients (29). However, this study also acknowledged significant challenges: the physiological myocardial PET signal uptake and the cardiac motion during PET imaging were major limiting factors in the study of coronary artery biology. Besides the difficulties of correcting for respiratory motion, cardiac contraction and myocardial uptake, even with suppression protocols the relative size of coronary arteries to voxel size greatly increased the risks of partial volume effects biasing measurements (30).

Engineering and workflow solutions to overcome these limitations were developed—targeting dietary preparation (low carbohydrate/high fat meals) (31–33), delayed imaging (4, 34), high-count iterative reconstruction (35, 36), and motion correction (37–41). Feasibility was shown to improve with specialised protocols as further tested by Cheng et al. in 2012, but still failed to detect an increased signal at the site of culprit lesions treated with PCI in nearly half of cases (42). Demeure et al. used this protocol in a randomised trial and showed that a high fat, low carbohydrate diet followed by a 12-hour fast did indeed suppress myocardial ^18^F-FDG uptake in most subjects and that a circulating free fatty acid level of 0.65 mmol/L was the best cutoff to predict adequate ^18^F-FDG uptake suppression (receiver-operating-characteristic curve analysis positive predictive value 89%) (43).

## Transition to sodium fluoride and microcalcification imaging

Sodium fluoride labelled with fluorine-18 (^18^F-NaF) has emerged in recent years as a tracer capable of overcoming the limitations of FDG in the study of coronary atherosclerosis. ^18^F-NaF binds exposed hydroxyapatite with high surface-area preference (44) (the fluoride ion exchanges with hydroxyl groups in hydroxyapatite [Ca₁₀(PO₄)₆(OH)₂]), making it a reporter of active microcalcification rather than bulk, mature calcium allowing evaluation of disease activity that cannot be captured by CT calcium scoring or standard CT coronary angiography. Microcalcification (<50*μ*m) – that is invisible in CT – has been implicated in cell death, necrosis and inflammation (45, 46). ^18^F-NaF additionally has preference for hydroxyapatite compared to other clinical salts (47).

The largest histological validation study of ^1^⁸F-NaF was conducted by Youn et al. (48) (Table 2) and included 101 plaques from 18 human coronary arteries that were assessed *ex vivo* with ^1^⁸F-NaF PET imaging. The investigators found a higher ^1^⁸F-NaF PET uptake in atherosclerotic plaques with microcalcification compared with no microcalcification [mean target to background (TBR) ratio ± SD, 9.0 ± 9.7, vs 2.9 ± 3.8; *P* < 0.0001]. 18F-NaF PET activity was higher in advanced plaques (fibroatheromas, *n* = 54, 10.7 ± 10.3), than in plaques with intimal thickening (*n* = 22, 3.5 ± 3.9) or pathological intimal thickening (*n* = 25, 6.1 ± 8.4; *p* = 0.004). Notably, in this study, 18F-NaF PET activity was significantly associated with microcalcification, whereas the association with CD68 staining for macrophages did not reach statistical significance (*P* = 0.08).

In clinical studies, coronary ^18^F-NaF uptake has been shown to be associated with high-risk features of coronary atheroma on CT coronary angiography (spotty calcium, positive remodeling) (9, 49, 50), while the study of Dweck et al. (8) that compared the performance of ^18^F-NaF and FDG showed that in contrast to FDG the coronary uptake of ^18^F-NaF is not masked by the myocardial activity due to differences in mechanism of action of the tracers with naturally low background NaF activity in myocardium. The same group in a prospective study of 80 patients (9) (40 with an ACS and 40 with stable coronary artery disease) showing that 93% of ACS patients demonstrated focal ^1^⁸F-NaF uptake in the culprit lesion, whereas 45% of the stable patients had a focal coronary activity. In that report ^1^⁸F-NaF uptake co-localised with high-risk plaque features on IVUS (positive remodelling, spotty calcification and low attenuated plaques), but not with dense calcification. In contrast, ^1^⁸F-FDG failed to identify culprit lesions due to myocardial background activity. This study demonstrated that ^1^⁸F-NaF PET could potentially non-invasively identify ruptured and high-risk plaques *in vivo*, serving as a marker of plaque vulnerability beyond conventional CT and offering biological and technical advantages over FDG. Interestingly this study also found that the ^1^⁸F-NaF PET counts were paradoxically higher in stable angina plaques than in plaques of patients with recent ACS, a finding that the authors attributed to the advanced age of the stable angina patients and the high-risk factor profiles. More recent reports have showed an increased ^1^⁸F-NaF PET activity in patients with rapid atherosclerosis progression and calcification formation (51) and that coronary microcalcification activity as assessed by ^1^⁸F-NaF PET did not fluctuate over the course of 12 months significantly (52).

The PRE^18^FFIR (Prediction of Recurrent Events With ^18^F-Fluoride Imaging, NCT 02278211) study was the first prospective study designed to test whether coronary ^1^⁸F-NaF PET activity could predict future cardiovascular events - myocardial infarction and cardiac death - beyond standard anatomical and clinical risk factors. This multicentre observational trial enrolled 704 patients with recent myocardial infarction who underwent coronary ^1^⁸F-NaF PET/CT and coronary CT angiography in a median of 20 days after their index event. The investigators measured the intensity and volume of ^1^⁸F-NaF uptake across each coronary artery and classify these as vessels with increased or no activity. At a median follow-up of 4 years [IQR 3-5 years], a global ^1^⁸F-NaF activity was not a predictor of the primary composite outcome including cardiac death non-fatal myocardial infarction or unscheduled revascularization [hazard ratio (HR), 1.25; 95% CI: 0.89-1.76; *P* = 0.20] but was associated with the secondary end-points cardiac death and myocardial infarction (HR: 1.82; 95% CI: 1.07-3.10; *P* = 0.03) and all-cause mortality (HR: 2.43; 95% CI: 1.15-5.12; *P* = 0.02) (53). Vessel-level analysis showed that an increase PET activity was associated with myocardial infarction in the same coronary territory (HR 2.08, 95%CI: 1.16–3.72; *p* = 0.013) (54). This effect appeared to be modified by treatment status: with worse outcomes noted in segments left untreated compared to those receiving a stent (HR: 3.86, 95%CI: 1.63–9.10 vs HR 1.02, 95%CI: 0.47–2.25; p for interaction=0.024).Vessel-level analysis also showed that the presence of multiple arteries with increased ^1^⁸F-NaF activity was associated with a higher risk of cardiac death or MI (HR: 2.43, 95%CI: 1.37–4.30; *p* = 0.002) and more MIs (HR: 2.50, 95%CI: 1.42–4.39; *p* = 0.002). These findings underscored the predictive value ^1^⁸F-NaF PET imaging but also reveal significant limitations of the tracer in detecting high-risk patients. There are several possible reasons for this which are likely to be additive. Coronary “activity” in PRE^1^⁸FFIR was defined as aggregate of ^1^⁸F-NaF distribution across a vessel rather than discrete focal uptake, a highly reproducible (55) but spatially diffuse metric that dilutes lesion-level specificity. Technical limitations—cardiac motion, partial-volume effects, and blood-pool spill-in—remain problematic even with dual (cardiac and respiratory) gating, making precise co-localisation of uptake within an individual plaque challenging. The signal itself is temporally stable: longitudinal data show little short-term change in ^1^⁸F-NaF activity over months, implying that it reflects chronic mineral metabolism rather than imminent plaque destabilization. Moreover, biologically ^1^⁸F-NaF detects surface hydroxyapatite deposition, a process that may represent both injury and healing, as microcalcification formation can mark either plaque progression or stabilisation depending on local inflammation and therapy and in particular statin exposure. This biological ambiguity, coupled with intensive secondary prevention treatment in contemporary cohorts, likely attenuates the efficacy of ^1^⁸F-NaF to detect vulnerable plaques and predict events.

In summary, ^1^⁸F-NaF offers a reproducible, biologically specific window into active coronary mineral deposition and retains prognostic value at the patient- and untreated-vessel level. However, its limitations in spatial precision, temporal responsiveness, and mechanistic specificity constrain its utility for identifying the imminently high-risk plaque. Taken together, the available evidence suggests that ^1^⁸F-NaF is more appropriately interpreted as a marker of plaque biological activity and active microcalcification than as a specific marker of plaque vulnerability or imminent rupture. Thus, exploration of alternative molecular targets that can more directly capture the inflammatory and destabilising biology – such as activated macrophages and hypoxia – which are involved in the formation of vulnerable plaques and their rupture is essential.

## Tracers targeting macrophage activation and inflammation

The central role of macrophages in atherogenesis and plaque destabilisation has made them a key target for molecular imaging. Early efforts employed metabolic tracers targeting cellular metabolism rather than receptor expression, building on the observation that activated macrophages exhibit high rates of membrane synthesis, lipid metabolism, and fatty acid oxidation. These tracers, while not cell-specific, provided some of the first insights into inflammatory plaque biology beyond FDG.

Kato et al. first reported incidental arterial ^11^C-choline uptake in an oncologic PET/CT 93 adults, noting that this occurred predominantly in older men and was frequently independent of calcification on CT (co-localised in just 6% of cases), suggesting detection of an alternative biological process (56). Laitinen et al. confirmed in LDLR⁻/⁻ApoB^1^⁰⁰/^1^⁰⁰ mice that ^11^C-choline uptake was almost twice that of controls (plaque-to-normal wall ratio 2.3 ± 0.6, *p* = 0.014) and colocalised with CD68-positive macrophages on autoradiography and immunohistochemistry (57). Further evidence came from Derlin et al., who found focal arterial ^11^C-acetate uptake in 32 out of the 36 patients (89%) with atherosclerotic vessels undergoing PET-CT imaging for malignancy; in that study only 29% of tracer-avid sites showed corresponding CT calcium, implying that acetate identifies metabolically active, non-calcified lesions (58). Finally, Vöö et al. studied 10 patients with symptomatic carotid stenosis and demonstrated higher ^1^⁸F-fluorocholine uptake in the culprit than the contralateral arteries [TBRₘₐₓ 2.0 [1.5–2.5] vs 1.4 [1.3–1.6]; *p* = 0.047], with the tracer activity correlating with macrophage density by CD68 staining (*ρ* = 0.65, *p* < 0.05) (59). The findings of these studies underscored the potential of choline- and acetate-based PET tracers in detecting metabolically active, macrophage-rich plaques. These tracers offer advantages over FDG as they are associated with lower myocardial background uptake (choline) and rapid blood clearance (both). However, the fact that these tracers are not exclusive to macrophages, and they have a short half-life have restricted their clinical utility, leaving them primarily as proof-of-concept demonstrations of how macrophage metabolism might be visualised *in vivo*.

The identification of surface receptors more specific to activated (M1-like) macrophages offered a promising alternative for assessing plaque inflammation. The radioligand ^11^C-PK11195 binds with high affinity and selectivity to the peripheral benzodiazepine receptor - now recognised as the translocator protein (TSPO) - which is abundantly expressed on activated macrophages. In humans, ^11^C-PK11195 has been successfully applied for *in vivo* imaging of neuroinflammation, providing a foundation for its later use in vascular imaging (60). A key early clinical demonstration of macrophage-specific molecular imaging came from Pugliese et al. (61), who used the translocator-protein ligand ^11^C-PK11195 to visualise vascular inflammation in patients with active vasculitis. In this study that included 15 patients an increased arterial wall tracer uptake (aorta or carotid) was noted in 6 symptomatic patients with vasculitis but none in the 9 asymptomatic controls [mean tissue to background ratio (TBR) 2.41 ± 1.59 vs 0.98 ± 0.10; *p* = 0.001]. Gaemperli et al. further validated PK11195 in 32 patients with carotid stenosis (62). Symptomatic plaques showed higher TBR than asymptomatic plaques (1.06 ± 0.20 vs 0.86 ± 0.11; *p* = 0.001) and lower CT attenuation. PET signal co-localised with CD68/TSPO in endarterectomy tissue (8 patients), and combining TBR with CT attenuation achieved 100% PPV for recent symptoms. Immunohistochemistry confirmed co-localisation of PK11195 binding with CD68-positive macrophages, providing the first direct human evidence that a macrophage-targeted PET tracer could detect vascular inflammation *in vivo*. Although limited by the short half-life of ^11^C and relatively high non-specific binding, this study established TSPO-based PET as an early proof-of-concept for selective macrophage imaging in atherosclerosis.

Another ligand of interest is somatostatin receptor subtype-2 (SSTR2) which is highly expressed on pro-inflammatory macrophages within human atheroma, providing a target for radiolabelled somatostatin analogues such as ⁶⁸Ga-DOTATATE and ⁶⁴Cu-DOTATATE. Preclinical studies confirmed that somatostatin receptor tracers accumulate selectively within inflamed, macrophage-rich arterial lesions. Li et al. demonstrated that ⁶⁸Ga-DOTATATE localises to atherosclerotic plaques in ApoE⁻/⁻ mice with *ex vivo* autoradiography showing focal uptake that co-localised with CD68-positive macrophages on immunohistochemistry (63). Receptor specificity was confirmed by competitive blocking with an anti-SSTR2 antibody and by parallel upregulation of SSTR2 on isolated macrophages. Pedersen et al. extended these findings to human carotid atheroma, showing that ⁶⁴Cu-DOTATATE uptake on PET/magnetic resonance imaging (MRI) correlated with macrophage gene markers within excised endarterectomy tissue, particularly with CD163 and CD68 expression (64). Together, these data provided strong histological validation that somatostatin receptor–targeted radioligands bind specifically to activated macrophages within inflamed arterial plaques, establishing the biological rationale for subsequent clinical imaging studies in human atherosclerosis.

Tarkin et al. performed the first clinical evaluation (10), directly comparing ⁶⁸Ga-DOTATATE and ^1^⁸F-FDG PET/CT in patients with carotid disease and coronary atherosclerosis. In 42 subjects, ⁶⁸Ga-DOTATATE uptake correlated strongly with macrophage density on histology (r = 0.79, *p* < 0.001) and identified culprit or high-risk plaques with greater target-to-background contrast than FDG, particularly in the coronaries where myocardial FDG activity precluded interpretation in 64% of cases. ⁶⁸Ga-DOTATATE signal was higher in culprit versus non-culprit lesions (TBR: 2.3 ± 0.5 vs 1.8 ± 0.4, *p* < 0.01), establishing somatostatin receptor imaging as a practical method for visualising arterial inflammation *in vivo*. These findings were not confirmed by the study of Wan et al. (65). The investigators prospectively recruited 20 patients with symptomatic carotid stenosis and performed ^68^Ga-DOTATATE PET/CT imaging before endarterectomy. Although image quantification showed good inter/intra-observer reproducibility (ICC generally >0.8 for SUVs), tracer uptake in symptomatic plaques was not significantly different from contralateral carotids, while immunohistochemistry failed to demonstrate SSTR2 expression on plaque macrophages. The authors concluded that ^68^Ga-DOTATATE activity observed *in vivo* did not reflect specific SSTR2-mediated binding in this cohort, arguing against a clinical role for SSTR2 PET for detecting symptomatic carotid plaques.

These studies illustrate both the potential and the current uncertainty surrounding somatostatin receptor imaging of vascular inflammation. The apparently contradictory findings of the study of Tarkin et al. and Wan et al. may be due to methodological differences between the studies, to differences in patient selection, the vascular territory studied, the timing of imaging relative to symptoms, the quantification, and the approach used for histological validation of SSTR2 expression. Considering this, SSTR2-targeted PET should currently be regarded as investigational in atherosclerosis.

The effective spatial resolution of PET—on the order of several millimetres—is large relative to the thickness of the inflamed intima, and even minor motion or mis-registration between PET and CT can obscure or falsely amplify signal. Moreover, receptor expression in plaques may vary with local milieu, therapy, or stage of disease, further complicating interpretation. As a result, the true biological specificity of SSTR2 uptake in atherosclerosis remains uncertain; further studies using improved motion correction, higher-resolution systems, and histological correlation are needed to clarify whether somatostatin receptor PET can reliably identify inflamed or high-risk plaques.

## Emerging pet targets for atherosclerosis

Over the recent years efforts have been made to developed novel tracers that will allow accurate characterization of vascular biology and detection of vulnerable plaques with a high sensitivity and specificity. The most promising are described below.

***Oxidation-specific epitopes (OSE): ⁸⁹Zr-LA25 (human antibody Fab)***: Targeting malondialdehyde–acetaldehyde adducts on oxidised lipoproteins offers a route to image lipid peroxidation within plaque. In rabbits and ex-vivo human carotid endarterectomy tissue, ⁸⁹Zr-LA25 PET/MR showed specific arterial uptake and correlating with OSE-rich lesions – supporting feasibility for non-invasive phenotyping of high-risk plaque biology (66). This tracer has not undergone first in man studies yet.
***Chemokine receptors (monocyte/macrophage trafficking):*** Several tracers have been introduced to detect the presence of chemokines in atherosclerotic plaques. The most important are described in this section:
*CCR2 (ECL1i peptide)*: CCR2 is implicated in monocyte recruitment in plaques and acceleration of lesion growth (67). Preclinical work established ⁶⁴Cu/⁶⁸Ga-DOTA-ECL1i as a highly specific probe for CCR2⁺ monocytes/macrophages, with competitive blocking, flow-cytometry validation, and cross-species binding (68). Translation to atherosclerosis studies is a logical next step given the pathway’s role in vulnerable plaque formation.
*CCR5 (DAPTA-decorated nanoparticle)*: CCR5 participates in leukocyte recruitment within plaques with a role in later disease and inflammatory tone (69). In ApoE⁻/⁻ vascular-injury–accelerated atherosclerosis, a CCR5-targeted PET nanoparticle imaged receptor expression and tracked biology relevant to progression/regression (70, 71).
*CXCR4:* CXCR4 regulates leukocyte and progenitor trafficking in plaques (72). Li X et al. evaluated (73) the CXCR4-targeted tracer ⁶⁸Ga-Pentixafor in 38 patients undergoing oncologic PET/MRI. Arterial uptake was observed in the aorta, carotid, and femoral arteries, with mean TBR 1.9 ± 0.3 in high risk-factor positive subjects versus 1.7 ± 0.2 in those without risk factors (*p* < 0.05). Tracer's uptake colocalised with MRI-defined atherosclerotic plaques. Immunohistochemistry of excised human carotid tissue (6 plaques from 3 patients) confirmed the CXCR4 expression on inflamed intima with CD68-positive macrophages. This study established the feasibility of imaging vascular CXCR4 expression in humans indicating that CXCR4-PET can be used as an alternative to FDG or SSTR2 signalling for detecting vascular activity.
***Folate receptor-β (FR-β) on activated macrophages.****^1^⁸F-FOL:* In mice and rabbits with atherosclerosis, ^1^⁸F-FOL PET/CT localised to FR-*β*–positive macrophage regions with plaque-to-background ratio comparable to FDG but with a far lower myocardial activity – an attractive property for coronary plaque imaging (74). Clinical atherosclerotic studies in humans with this tracer have not been performed yet.
***Endothelial activation/adhesion.*** *VCAM-1 ligand ^1^⁸F-4V:* VCAM-1 is an inducible endothelial adhesion molecule which binds leukocyte *α*4*β*1 to mediate firm adhesion and transendothelial migration, amplifying endothelial inflammatory signalling. A prototypical endothelial-inflammation tracer, ^1^⁸F-4 V enabled PET-CT detection of VCAM-1 in atherosclerotic plaques in ApoE⁻/⁻ mice (75). Uptake co-localised with Oil Red O and correlated with VCAM-1 mRNA (R = 0.79; *p* = 0.03). In this study, atorvastatin reduced lesional signal, demonstrating treatment responsiveness. This tracer appears to map an upstream inflammator*y* axis not captured by other tracers.
***Vascular adhesion protein-1 (VAP-1).*** *⁶⁸Ga-DOTA-Siglec-9:* VAP-1 is an endothelial adhesion molecule that is upregulated on inflamed endothelium, facilitates leukocyte recruitment (76), and can be targeted by ⁶⁸Ga-DOTA-Siglec-9. First-in-human studies in healthy volunteers showed favourable biodistribution and dosimetry (77), while preclinical studies in atherosclerotic mice reported plaque uptake that co-localised with VAP-1–positive endothelium and macrophage-rich lesions (78, 79). Early applications of this tracer in pre-clinical myocarditis models and patients with giant cell arteritis supports its clinical potential – however its role in the study of atherosclerosis remains elusive.
***Angiogenesis.*** *αvβ3-integrin RGD peptides*: *α*v*β*3 integrin is upregulated on angiogenic endothelium, macrophages, and vascular smooth muscle in plaques. Cyclised RGD peptides have affinity and selectivity for *α*v*β*3 integrin with ⁶⁸Ga-NODAGA-RGD offering a method for detecting tissue expression of integrin *α*v*β*3 *in vivo* (80). In 44 adults (8 with previous atherosclerotic disease; 36 controls), arterial ⁶⁸Ga-NODAGA-RGD uptake was higher in those with atherosclerotic disease [mean TBR 2.44 (2.03–2.55) vs 1.81 (1.56–1.96); *p* = 0.001]; tracer uptake was correlated with prior cardiovascular and cerebrovascular events (r = 0.33; *p* = 0.027) a high BMI (*ρ*=0.38; *p* = 0.01) an increased plaque burden (*ρ*=0.31; *p* = 0.04), and hypercholesterolaemia (r = 0.31; *p* = 0.04) (81). A more recent study with ^18^F-fluciclatide PET tracer for *α*v*β*3 integrin showed aortic signal associated with atheroma burden that was also higher in patients suffering a myocardial infarction (TBR_max_ 1.29 vs 1.21; *p* = 0.02). These studies underscore its potential to detect angiogenesis in large arteries; coronary application has yet to be demonstrated.
***Macrophage nanoprobes.*** *⁶⁴Cu-Macrin (polyglucose/dextran nanoparticle)*: this is a ∼20-nm spherical dextran (polyglucose) nanoparticle labelled with ⁶⁴Cu that accumulates in tissue macrophages by phagocytosis. In mice, rabbits, and pigs, ⁶⁴Cu-Macrin PET/MR visualised macrophage-rich inflammation in infarcted myocardium, inflamed lung, and atherosclerotic aortic plaques, with ex-vivo autoradiography and immunohistochemistry supporting macrophage localization (82). A first-in-human study testing the feasibility of this tracer and its accumulation in patients with cancer, sarcoidosis or myocardial infarction is currently underway (NCT04843891).

## Discussion

**Figure 3:**
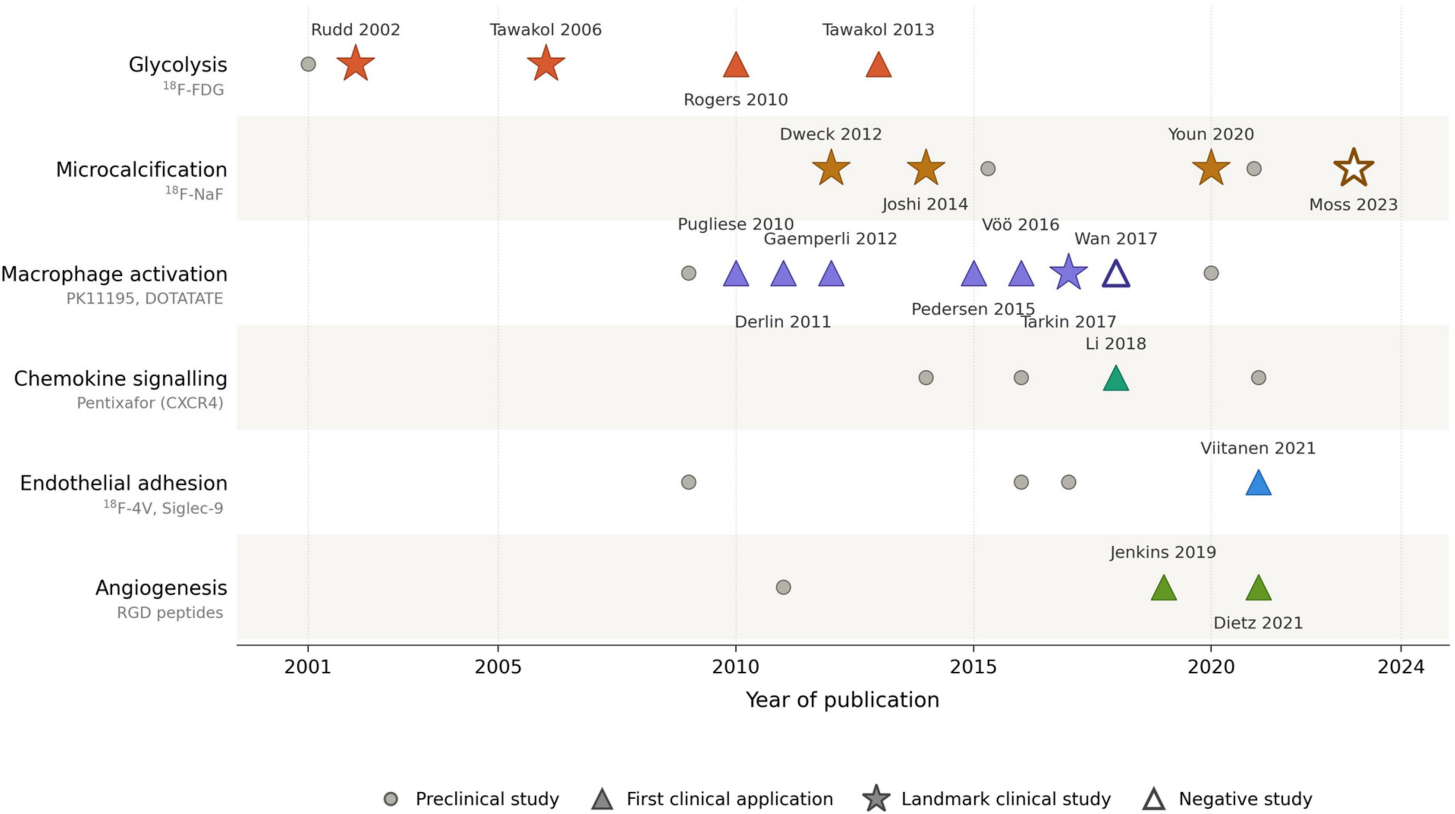
A chronological overview of PET tracer development for the *in-vivo* study of atherosclerotic plaque biology. Individual studies are plotted by year of publication (*x*-axis) and biological target (*y*-axis). Shape denotes study tier: filled circles represent preclinical studies, filled triangles represent first clinical applications, and filled stars represent landmark clinical studies (defined as large histological validation cohorts, first-in-human prospective trials, or outcome studies linking tracer activity to major adverse cardiovascular events). Open shapes denote negative clinical studies. The prototypical PET tracer for each biological target is listed beneath the corresponding *y*-axis label.

Over the last two decades PET has progressively expanded beyond glucose metabolism to deliver biological readouts spanning inflammatory cell activation, active mineral deposition, endothelial adhesion and chemokine signalling, revealing mechanistic pathways of atherosclerosis that precede or accompany structural changes (Figure 3). Among non-invasive imaging techniques that employ radioactive tracers to interrogate plaque biology, PET has emerged as the most developed platform for *in-vivo* assessment of vascular biology in atherosclerosis. Its higher spatial resolution and sensitivity relative to single-photon emission computed tomography, its capacity for accurate quantification of tracer counts, and the availability of line-of-response-based attenuation correction together render PET the preferred modality for interrogating the small-calibre, dynamically moving coronary artery wall. Despite this progress, translation of vascular PET into routine clinical application remains constrained by several technical limitations. These relate principally to spatial resolution, motion-related degradation and co-registration challenges.

### Spatial resolution

Spatial resolution remains a major limitation for PET imaging of atherosclerosis. Even on state-of-the-art systems the effective clinical spatial resolution remains on the order of ∼3.5–6 mm (worsening off-axis), which is large relative to the sub-millimetre arterial wall and the patchy distribution of macrophages or microcalcification - ushering in partial-volume loss and spill-in from blood pool and myocardium. Representative performance figures report ∼3.5 mm at isocenter for modern digital SiPM systems, degrading to ∼5–8 mm with radial offset typical of the heart, underscoring why lesion-level reads are so challenging without post-processing and careful quantification.

### Motion correction

The increased time required for imaging generates the need for further post-processing of the collected data especially in the coronary arteries where elimination of the effect of the cardiac and respiratory motion blur is essential for meaningful quantitative analyses. Dual gating of the collected PET signal in this setting and non-rigid motion correction (especially with PET/MR) improves sharpness and TBR but they add complexity, reduce counts if simply gated, and have yet to be standardised across platforms. Considerable efforts have been made in recent years to improve co-registration of coronary PET with anatomical imaging through motion-correction approaches. Several methodologies have been proposed that are based on cardiac and respiratory gating, non-rigid motion fields derived from CT or MR imaging, and data-driven frameworks (37, 83–86). MR-based motion fields (83–86) and data-driven gating (87–89) can recover contrast and improve localization for both FDG and NaF (and newer tracers), and targeted reconstructions (small voxels, point-spread-function modelling, time-of-flight) (90–92) can further mitigate the partial-volume loss; these steps are steadily being folded into cardiovascular PET workflows. Although these approaches appear to improve image sharpness and target-to-background ratios, extensive validation of their accuracy using established reference standards or of their clinical value for coronary plaque localisation remains limited.

The advent of long-axial-field-of-view (LAFOV) and total-body PET systems offers order-of-magnitude sensitivity gains that may help address some of PET limitations. That sensitivity can be leveraged to deliver finer voxels, longer list-mode acquisitions for robust dual (cardiac + respiratory) motion correction, and low-dose dynamic protocols - each directly relevant to coronary plaque imaging. Early clinical platforms (e.g., EXPLORER) have demonstrated whole-body dynamic imaging with 1-second temporal sampling (1 frame per second), enabling new kinetic strategies; the technology roadmap points to further gains in timing resolution and depth-of-interaction that could ultimately push effective resolution toward ∼2 mm in clinical systems.

PET has shown that plaque activity is dynamic and often diffuse at the vessel and patient level; lesion-scale forecasting remains hard because (i) our biological targets (macrophages, microcalcification) do not map 1:1 to imminent rupture, (ii) signals can represent injury or healing depending on context, and (iii) therapy rapidly modifies the substrate we image. These realities argue for humility in interpreting single-lesion hotspots and justify the diversification of tracers—from NaF to SSTR2, CXCR4, TSPO, FR-*β*, VCAM-1, VAP-1 and beyond—because no single marker can capture the full, shifting phenotype of the “vulnerable” plaque.

At present, no PET-based plaque imaging strategy is ready for routine clinical use in the assessment of atherosclerotic plaque vulnerability. Although FDG and ^1^⁸F-NaF have provided important mechanistic and prognostic insights, evidence remains insufficient to support their integration into routine clinical workflows for treatment selection or lesion-specific risk stratification. Important technical challenges including the standardisation of acquisition and PET quantification have not been addressed and there is lack of robust validation of motion-correction and co-registration methods for plaque localisation. In particular, harmonisation of quantitative readouts — encompassing TBR, absolute SUV, and emerging kinetic indices — is required to enable meaningful cross-study comparison and to support eventual clinical translation. Moreover, there are concerns related to the cost and additional radiation and absence of evidence showing that PET-guided assessment changes management and improves clinical outcomes; that would justify the routine use of PET imaging in clinical practice.

Nevertheless the future is promising. PET has already expanded our field of view from anatomy to biology and with LAFOV/total-body scanners, better motion correction, and smarter reconstructions is expected to enable precise quantification of vessel biology at a lesion level. Until clinical systems achieve consistently sub-3-mm effective resolution in the heart and standardised motion-corrected pipelines, lesion-level risk assignment will remain challenging and we should limit its use to patient-level risk stratification. However, ongoing developments in the field are anticipated to reduce radiation dose, enable high-quality co-registration of anatomical imaging from the CT and of several bio-pathological processes using complementary PET tracers and enable accurate detection of vulnerable plaques. PET constitutes the only non-invasive modality with this molecular reach; it is therefore essential to invest in this modality overcome the existing barriers and unlock its full potential in the study of coronary atherosclerosis.
